# Reconceptualizing the impostor phenomenon as an occupational experience: a scoping review and framework synthesis of qualitative evidence guided by the model of human occupation

**DOI:** 10.3389/fpubh.2026.1975155

**Published:** 2026-10-21

**Authors:** Latika Pandey, Fathima Yasmin, Rinshidha Gibin, Mehar Fathima, Suman Jabeen, Asniyah Sharieff, Junaid KTK, Ranajit Das

**Affiliations:** 1 Department of Occupational Therapy, Yenepoya School of Allied Health Sciences, Yenepoya (Deemed to be University), Mangalore, Karnataka, India; 2 Centre for Systems Biology and Molecular Medicine, Yenepoya Research Center, Yenepoya (Deemed to be Univesity), Mangalore, Karnataka, India; 3 Centre for Integrative Research in Clinical Genetics and Metabolic Medicine, Yenepoya Medical College and Hospital, Yenepoya (Deemed to be University), Mangalore, Karnataka, India

**Keywords:** healthcare professionals, impostor phenomenon, model of human occupation, occupational adaptation, occupational functioning, occupational participation, occupational performance, professional identity

## Abstract

**Background:**

The Impostor phenomenon is characterized by persistent feelings of self-doubt and difficulty internalizing accomplishment despite being competent. Although its psychological consequences among healthcare professionals have been extensively studied, the influences of impostor phenomenon on occupational functioning remains underexplored. This scoping review synthesized qualitative evidence to examine the experiences of healthcare professionals with impostor phenomenon through the Model of Human Occupation (MOHO), providing an occupational science perspective on impostor phenomenon.

**Methods:**

A scoping review was conducted in accordance with the Joanna Briggs Institute approach and reported following the PRISMA extension for Scoping Reviews. The protocol was pre-registered on the Open Science Framework. PubMed/MEDLINE, ProQuest Central, and Web of Science databases were searched for English-language qualitative studies published between 2015 and 2025. Data were charted and a framework synthesis was conducted using the Framework Method, with MOHO as the *a priori* analytical framework.

**Results:**

Eleven studies met the inclusion criteria. Within volition, participants demonstrated a competence-confidence disconnect, attributional challenges and gradual reconstruction of perceived competence, while values remained largely intact despite self-doubt. Habituation reflected disrupted professional identity formation, role transitions and compensatory occupational patterns including concealment, overpreparation and perfectionistic work routines. Performance capacity was characterized by altered subjective experiences of occupational performance, including emotional distress, cognitive burden and perceived inadequacy despite evidence of competence, with consequences for learning, participation and performance. Environmental influences included supportive mentorship, constructive feedback and psychologically safe workplaces, whereas hierarchical cultures, discrimination, workload pressures and organizational expectations could potentially reinforce IP.

**Conclusion:**

Interpreted through MOHO, the findings suggest that IP may be experienced across interconnected occupational processes, encompassing perceived competence, and professional role development, patterns of doing and influence of the environment. These interpretations require further cross-professional and longitudinal evidence. This review highlights the value of occupation-centered approaches for understanding and addressing IP and identifies opportunities for occupational therapy, healthcare education and organizational interventions that support healthy professional development.

**Systematic review registration:**

OSF, registration DOI: https://osf.io/xrbpd

## 1 Introduction

The Impostor Phenomenon (IP) is characterized by persistent feelings of inadequacy and self-doubt experienced by high-achieving people who are unable to internalize their accomplishment despite significant external evidence of their competence (1). Individuals often experience anxiety, self-doubt and feelings of alienation in an environment where their abilities, performance and competence may be constantly evaluated (1, 2). A systematic review and meta-analysis revealed approximately 62% prevalence of IP worldwide among health workers (3). IP has a noticeable impact on healthcare professionals as it is associated mental health outcomes, particularly compassion fatigue, burnout, low self-esteem, anxiety, and depressive symptoms (3–5). The paradox of IP is that individuals frequently attribute their successes to outside forces like luck rather than acknowledging their own skills, which represent the cycle of self-doubt and discontent at work (1, 2, 5).

Within occupational therapy, occupations extend beyond employment or work-related activities and refer to the everyday activities that people engage in individually, with families and within communities to occupy time and bring meaning and purpose to life. Occupations encompass activities that people need to do, want to do and are expected to do (6). Occupations influence how individuals experience themselves through doing, being, belonging, and becoming, and may contribute to experiences of identity, competence, belonging and purpose (7). In this review, the term occupational experience refers to how IP is situated within and expressed through the occupations of healthcare professionals. This encompasses not only professional roles and routines, but also other meaningful occupations and patterns of participation that may intersect with professional life, extending the reconceptualization of IP beyond work experience or professional functioning to consider how individuals experience, interpret and engage in occupations.

Although the psychological consequences of IP have been extensively investigated, existing research has predominantly focused on emotional distress, personality traits and mental health outcomes (3, 5, 8–12); however, less attention has been given to how impostor experiences are situated within everyday occupations, including role development, occupational routines, participation in meaningful activities and influence of workplace environment and context. When IP is primarily conceptualized as a psychological phenomenon, it remains underexplored how experiences such as self-doubt, perceived inadequacy and fear of failure are expressed through what healthcare professionals do, how they enact and develop their roles, how they organize their routines and how these experiences interact with the occupational environments where they practice. An occupational perspective may therefore complement existing psychological conceptualizations by exploring how these experiences are embedded within occupational becoming, being and functioning as a healthcare professional. The Model of Human Occupation (MOHO) was established to explore, organize and make explicit the concept of human occupation by supporting practice that is occupation-focused, client-centered and evidence-based (13). It provides a useful framework for addressing this gap as it may help understand how the IP affects an individual’s daily life, motivation, and ability to function in their roles and occupations. Despite being a psychological experience, IP is associated with behaviors that may disrupt performance of everyday activities. Within MOHO, four interrelated components help explain this process: (1) Volition refers to an individual’s pattern of thoughts and feelings about themselves and the activities they engage in, including their motivation, values, interest, and sense of competence; (2) Habituation illustrates the internal readiness to carryout consistent behaviors through habits and roles that are shaped by daily routines, time, place, and social expectations; (3) Performance capacity explains the individual’s ability to perform tasks, including both their actual abilities and their confidence in carrying them out; and (4) Environment includes the physical, social, cultural, economic, and political aspects of an individual’s context that influences the first three components (13). Therefore, MOHO provides a framework for exploring IP beyond its psychological manifestations by considering the dynamic, interrelated nature of occupational participation and functioning related to perceptions of competence and how it may interact with established roles and routine, experiences of performing occupations within environmental contexts. This scoping review aimed to map and synthesize qualitative evidence on the experiences of healthcare professionals with the impostor phenomenon through the lens of MOHO. By interpreting qualitative findings across the domains of volition, habituation, performance capacity and environment, the review sought to develop an occupational understanding of how IP may influence occupational participation and performance within healthcare settings.

## 2 Methodology

This scoping review was conducted in accordance with the Joanna Briggs Institute (JBI) methodology for scoping reviews (14) and is reported following the Preferred Reporting Items for Systematic Reviews and Meta-Analyzes extension for Scoping Reviews (PRISMA-ScR) (15). The review protocol was pre-registered on the Open Science Framework (OSF) to enhance methodological transparency and reduce the risk of reporting bias. Deviations from the registered protocol were as follows: the analysis, planned as a deductive thematic analysis, was conducted as a framework synthesis using the Framework Method in response to peer review; Zotero was used alongside Mendeley for de-duplication; and the population was broadened to include residents, doctoral students and biomedical researchers.

Table 1 displays the eligibility criteria guided by the Population-Concept-Context (PCC) framework recommended by the JBI. It included qualitative studies and mixed-method studies with separately reported qualitative findings. The publication window was restricted to 2015–2025 to capture more contemporary qualitative evidence within the emerging and developing literature on IP. The search was restricted to English-language publications based on feasibility of screening, interpretation and synthesis within the review team; the potential for language bias is acknowledged as a limitation. Grey literature such as theses, reports and conference abstracts was excluded as it is often brief, non-peer reviewed and inconsistent in reporting, in order to focus on qualitative studies with sufficient methodological and contextual detail to support meaningful thematic analysis and mapping into MOHO. This approach may have, however, resulted in omission of relevant qualitative findings reported in grey literature particularly given the emerging nature of research on IP.

**Table 1:** Eligibility criteria based on the Population-Concept-Context (PCC) framework recommended by the JBI.

Serial no.	PCC element	Inclusion criteria	Description
1.	Population	Healthcare professionals	Studies involving any healthcare professionals, residents and doctoral student in professions including but not limited to doctors, nurses, dentists, allied health professionals, social workers, pharmacists, mental health workers, audiologists and speech therapists, biomedical professionals and researchers, etc.
2.	Concept	Impostor Phenomenon	Studied describing experiences of, perspectives and perceptions towards impostor phenomenon.
3.	Context	Healthcare settings, qualitative studies (2015–2025)	Qualitative studies, or mixed-method studies with separate qualitative analysis, among various healthcare practice settings including but not limited to hospitals, clinics, day care centers, rehabilitation centers, community settings, hospice care, research and academia.

A comprehensive JBI recommended three-step search process was conducted across three databases: PubMed/MEDLINE, ProQuest Central, and Web of Science. The databases were selected to provide complementary multidisciplinary coverage relevant to the review question. PubMed/MEDLINE was selected to capture literature concerning clinical and health-related contexts; ProQuest Central was used for its multidisciplinary coverage across health sciences, psychology, social sciences, and related fields; and Web of Science Core Collection was included to identify interdisciplinary literature spanning healthcare, behavioral and social sciences. Together, these databases were considered appropriate for identifying qualitative literature examining impostor experiences among healthcare professionals from psychological, professional, and contextual perspectives. Supplementary Table 1 reports the database-specific search strategies. The review protocol was developed from October 2025 and registered on the Open Science Framework in March 2026 following institutional review and approval. Electronic database searches were conducted in April 2026, followed by study selection, data extraction and framework synthesis until July 2026. Search terms combined keywords related to the impostor phenomenon, healthcare professionals, and qualitative research using appropriate Boolean operators (14). Databases specific search strategies were developed and adapted according to indexing terms and search syntax. A reference strings was: (“impostor syndrome” OR “imposter syndrome” OR “impostor phenomenon” OR “imposter phenomenon” OR impostorism OR “impostor feelings”) AND (“healthcare professionals” OR “health care professionals” OR “health personnel” OR “health professionals” OR physicians OR doctors OR nurses OR pharmacists OR dentists OR residents OR “allied health” OR therapists OR “occupational therapists” OR “physical therapists” OR “medical students” OR “nursing students” OR “allied health students”) AND (qualitative OR “qualitative research” OR interviews OR interview OR “focus group” OR “focus groups” OR phenomenology OR phenomenological OR “thematic analysis” OR “grounded theory” OR “content analysis” OR “mixed methods”).

All identified records were exported to citation managers (Mendeley, Zotero) for removal of duplicate citations. Titles and abstracts were screened and followed by full-text review of potentially relevant articles adhering to the PRISMA-ScR flow diagram (15) for study selection process as displayed in Figure 1. The reference lists of included articles were also screened to ensure comprehensive coverage.

**Figure 1:**
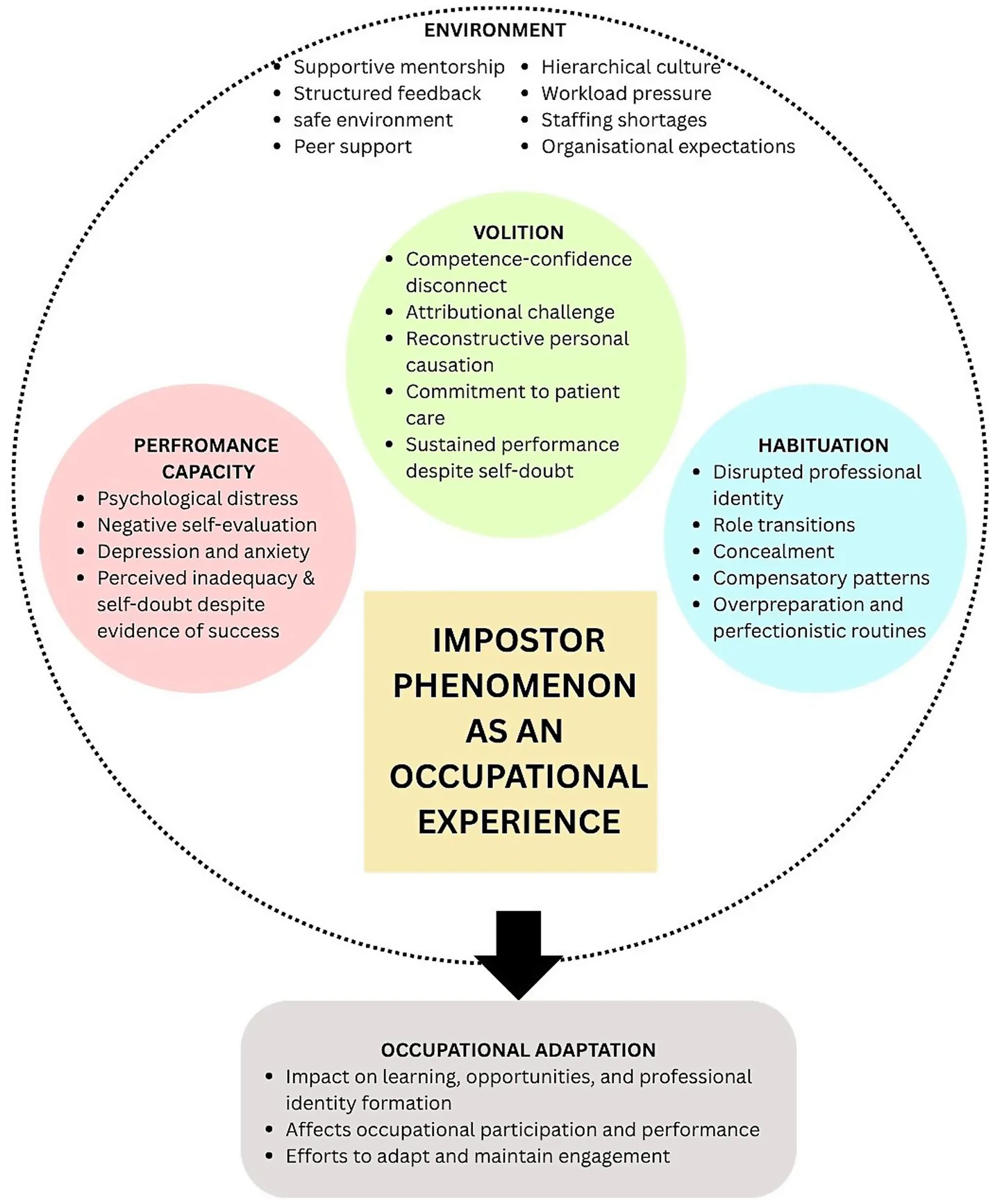
Study selection process presented using the PRISMA Extension for Scoping Reviews (PRISMA-ScR) flow diagram.

## 3 Data extraction

A data extraction tool was developed and first piloted on 3 studies to ensure clarity and relevance of items extracted. Supplementary Table 2 presents the country, profession, sample characteristics, methodology and key findings of the 11 included studies.

## 4 Results and data synthesis

Data were synthesized using framework synthesis (16), following the stages of the Framework Method (17), with MOHO serving as the *a priori* framework. As the included studies did not conceptualize impostor experiences using MOHO domains, participants’ experiences and meanings as reported in the original studies were first extracted in Supplementary Table 2. After familiarization with the extracted findings, the first seven authors independently coded each study’s findings to the MOHO domains and their components. Codes were charted manually into a study × component matrix for each domain, noting where evidence was indirect or absent. Supplementary Table 3 presents the Framework matrix charting of extracted findings to the MOHO domains of volition, habituation, performance capacity and environment. The matrices were then compared across studies to identify recurring patterns, variations and contradictions, which were subsequently developed into themes and subthemes through review team discussions until consensus was reached. To enhance credibility and trustworthiness of the synthesis, an external reviewer examined the matrices and themes. Results also report where evidence for a particular MOHO domain was limited or not directly identified. Similarly, other relevant findings that did not correspond to any particular theme but provided context for understanding the participants’ experiences were also retained and reported in the appropriate MOHO domains. Therefore, MOHO functioned as an organizing and interpretive framework rather than determining the final themes in advance; the themes were developed from comparison and synthesis of the reported qualitative findings within and across the MOHO domains.

### 4.1 Volition

#### 4.1.1 Theme 1: competent, yet not confident: a disruption of personal causation

Across the included studies, a recurring pattern of discrepancy between demonstrated competence and participants perceived competence, which was mapped to personal causation within MOHO. Three interconnected patterns were identified across studies: (1) A competence-confidence disconnect, (2) attributional challenges, and (3) gradual reconstruction of perceived competence.

##### 4.1.1.1 The competence-confidence disconnect

Across the studies participants described a recurring discrepancy between demonstrated competence and perceived competence. Early-career nurses, residents, simulation educators, researchers, and MD-PhD trainees reported progressing through occupational demands and professional challenges while continuing to question whether they possessed the ability required to occupy their roles (18–26). In the study by Ford et al. (18) a participant described the impostor experience to be *“*more of a confidence thing than a competence thing.” Despite completing professional training, managing clinical or research responsibilities, and receiving positive feedback, participants continued to question whether they were sufficiently capable or deserving of their roles. Mirabal et al. (19) captures the contradiction clearly as quoted “Rationally, I can look at my accomplishments… But then there’s still those thoughts and feelings… of self-doubt… I slip back into those feelings of inadequacy”. Participants struggled to believe positive feedback, evaluated their competence against more experienced or specialized peers, continued feeling unprepared to function at the level expected of them, and perceived themselves as underperforming (23). However, LaDonna et al. (27) interprets this discrepancy as repeated external questioning of ability, suggesting being devalued or judged against gender-based differential standards by others to be a contributing factor.

##### 4.1.1.2 Attributional challenge: success discounted, failure internalized

A second recurring pattern concerned how occupational experiences were interpreted. Evidence supporting competence was frequently discounted, while mistakes, uncertainty or unexpected outcomes were readily incorporated into participants’ perception of themselves as inadequate (19, 21, 23, 24, 28). Successful patient outcomes or achievements could be attributed to luck, while praise could be interpreted as resulting from misunderstanding or inaccurate judgment by the person providing it, as reflected in the response “it must have been some misunderstanding on the part of whichever person gave me praise.” (23) Gupta et al. (24) found that residents could disproportionately personalize mistakes until leaders helped them recognize systems-level contributions and shared responsibility for patient-care error, stated as “My attending took me aside when I was feeling bad about a mistake with a patient and talked me through the systems factors involved with mistakes in patient care, the Swiss cheese model, and the shared responsibility for all mistakes in patient care.” With such mentorship, they became less likely to automatically blame themselves. The environmental context may play an important role because external attribution was sometimes actively supplied by others such as comments towards minority status, repeated external questioning and differential treatment (22, 27).

##### 4.1.1.3 Reconstructing personal causation

Perceived competence was dynamic and could potentially change with experience and support. Across several studies, participant experiences, successful performance, feedback, supportive validation, recognition of accomplishments, and supported autonomy were described as opportunities for participants to develop a more realistic perception of their own capabilities (18–20, 24, 26, 28). Lubega et al. (28) found that accumulated experience, successful projects, troubleshooting, and supportive mentorship reduced automatic self-blame, and personal inadequacy, as stated, “As students progressed and had success in other projects, they became less likely to blame themselves when experiencing irreproducibility.” Interpersonal validation was identified as a prominent supportive factor. Ryan et al. (20), described successful patient-care experiences, mentor affirmation and positive patient feedback strengthened perceptions of competence as reflected in statements such as “…when you get that positive feedback from your patients…you know you’ve made a difference with them.” and “…You talk to someone else they’re feeling exactly the same way you are; it made a big difference” (20). Reconstruction could also involve changing the interpretation of the impostor experience itself. Reframing recurring IP, resistance against externally imposed messages of incompetence and retention of a sense of capability rather than internalizing discriminatory treatment as something that could motivate continued learning and growth (25, 27). Overall, findings suggested that self-directed negativity could interfere with learning and transformation, highlighting the importance of overcoming self-directed negative appraisals (21).

#### 4.1.2 Theme 2: values that induce meaning but raise the stakes

Unlike personal causation, values were less explored across the included studies. Two interconnected patterns were identified: (1) valuing meaningful professional contribution and (2) valuing professional competence, integrity and growth.

##### 4.1.2.1 Valuing meaningful professional contribution

Across the studies within healthcare context, participants expressed concern about providing competent care, fulfilling professional responsibilities and making a meaningful contribution to patients. Impostor experiences occurred alongside a strong sense of responsibility towards those receiving care Mirabal et al. (19) described residents’ experiences of inadequacy in conjunction with an *“incredible responsibility”* toward patients, suggesting that concerns about competence acquired greater significance when participants perceived themselves as responsible for patient outcomes. Similarly, Chodoff et al. (23) identified a strong sense of responsibility toward patients, which could extend into excessive personal responsibility for poor patient outcomes.

Among early-career nurses, patients were described as being central to participants’ work, with providing appropriate care and making a meaningful difference contributing to professional purpose (20). Positive patient feedback reinforced the meaning of work, with one participant reflecting that “you know you’ve made a difference.” (20) Similarly, Ford et al. (18) identified concern about meeting expectations for safe and appropriate patient care during the transition into qualified practice. Chow et al. (21) described trainees’ commitment to competent patient care and their desire to honor the trust placed in them by patients and mentors. Gupta et al. (24) described responsibility toward patients was particularly apparent in response to mistakes. Uncertainty about one’s own ability could become consequential and responsibility could extend into excessive personal accountability for outcomes, for example, Ryan et al. (20) reported that a participant stated “It was harder to deal with my own head than dealing with the patient… the feeling of inadequacy, being a new nurse, dealing with a patient that’s deteriorating, and feeling like an imposter… I cried.”

##### 4.1.2.2 Valuing professional competence, integrity and growth

A second pattern reflected the importance participants placed on becoming competent, legitimate and growing (23, 25, 28), although these values were less consistently reported than those relating to patient care. For instance, Freeman et al. (25) described a constant pursuit of credentials, reflected in the statement “We’re always chasing letters… we need to get the badge…” This suggests that IP did not necessarily reduce motivation towards professional growth but led to concerns about whether one had achieved sufficient competence. However, these expectations could become problematic when participants judged themselves against unrealistic standard of what they believe they should already know, as stated, “pressure of needing to know everything… realizing that’s not possible… always feeling like you are underperforming” (23).

Interests were rarely identified directly across the included studies; however, these could not necessarily be interpreted as interest within volition-MOHO. It included satisfaction being derived from meaningful patient interactions (20), and experiences of irreproducibility reducing participants’ motivation and enthusiasm for research, meanwhile successful troubleshooting and resolution of research difficulties restoring enthusiasm (28). A smaller group of findings suggested that professional values and aspirations could interact with sociocultural expectations, such as tension between MD-PhD participants career direction and family expectations, as reflected in statements “I wouldn’t do well because no one in my family was in medicine, and my undergraduate degree wasn’t competitive enough…” (22) and gendered expectations regarding how medical professionals should behave and legitimacy within particular medical roles (27). These findings suggest that meaningful occupational directions could also be shaped by external expectations and are reported further within the environmental domain.

### 4.2 Habituation

#### 4.2.1 Theme 3: becoming and navigating the professional roles

Two patterns emerged within professional roles: (1) increasing responsibility and transitioning roles and (2) navigating multiple and intersecting professional roles.

##### 4.2.1.1 Increasing responsibility and transitioning roles

Across studies in contexts of clinical training and early professional practice, transitions into new or more advanced roles frequently brought increased responsibility, autonomy and expectations of competence before participants experienced themselves as ready to meet those expectations (18–21, 23, 28). Newly qualified nurses transitioning from the supervised student role into independent registered nursing practice described continuing to experience themselves as students (18, 20). One participant captured this simply as “I still felt like a student…” suggesting that participants began performing as independent professionals before fully experiencing themselves as such (20). The shift required independent medication administration, clinical decision-making and responsibility for patient care without the immediate supervision that had previously structured their performance. Role-related uncertainty was not restricted to initial role transition. Increased expectations and responsibilities were associated with resident roles occurring alongside feelings of unpreparedness, as reflected in the statement, “You walk in and are expected to be able to put in orders…that you don’t feel [prepared for]…I think there is a lot more expectations and responsibilities as a resident, that you feel like an impostor because you may not have had [those] experiences yet. And yet you’re supposed to be competent and perform those duties” (19). The transition from intern to senior resident was noted as participants moved from seeking answers themselves, to becoming the person junior colleagues expected the answers from, as stated, “you transition from being an intern to a second year…new interns are turning to you for all the answers.” (23). Similar findings were described around increasing seniority that involved developing an identity as an independent cardiologist (21), and senior researchers (28) where IP persisted despite progression in training, whereas increasing experience and successful projects were associated with greater confidence.

##### 4.2.1.2 Navigating multiple and intersecting professional roles

A second pattern concerned participants who moved between or simultaneously occupied multiple professional roles, each carrying different expectations of competence (22, 24, 25, 27). Such as simulation educators who moved between clinical and educational identities and between positions as learners, teachers, facilitators and mentors (25), and MD-PhD trainees were required to negotiate both physician and scientist roles across medical and research environments (22). New responsibilities, projects or senior roles could reactivate doubts about legitimacy even among experienced professionals, suggesting that the challenge involved not simply becoming more senior but establishing legitimacy across different areas of professional practice. Frequent role transitions required adaptation to a different role interrupting continuity in professional identity development.

However, developing professional role could be reinforced through opportunities to enact the role and recognition from others, such as autonomy, entrusted with clinical decision-making and publicly recognized by senior clinicians as legitimate physicians (24). This suggests a counter process that role internalization could develop when increasing responsibility was accompanied by supported autonomy and recognition rather than expectation alone. Conversely, professional role legitimacy could be externally challenged, women physicians described gendered expectations, difficulty being taken seriously in positions of authority and pressure to modify their behavior to fit expected professional norms (27).

#### 4.2.2 Theme 4: patterns of doing in response to perceived inadequacy

Across the studies, impostor experiences were also associated with recurrent behavioral responses oriented toward managing the possibility that perceived inadequacy would become visible to others. Two related patterns emerged: (1) Concealing uncertainty to avoid exposing perceived incompetence and (2) increasing effort to compensate for it.

##### 4.2.2.1 Concealment and protective self-presentation

Several studies described recurrent attempts to hide uncertainty, vulnerability or perceived weakness (19, 22–25, 27). It was observed in the form of hesitancy to ask questions or seek help and pretending that they knew things rather than expose uncertainty (19, 23). One participant explained that feeling uncomfortable asking questions could “make me want to just say that I know things and maybe not reach out for help when I need it.” (19) Concealment could become recurrent rather than situational. One participant reported “burying this feeling on an almost daily basis” and hiding from others when actively experiencing IP (24). Participants becoming quieter, holding back contributions, avoiding visibility or opportunities and minimizing presence (17, 22). Behaviors such as self-monitoring, altered self-presentation and withholding participation in response to discriminatory professional environments were also identified (27).

##### 4.2.2.2 Doing more as compensatory effort

Another response involved increasing occupational effort to compensate for perceived inadequacy (19–21, 25, 27, 28). Overpreparation was identified to become embedded into everyday routines. One participant described arriving at “5:30 in the morning for pre-rounding” specifically because they were “compensating for my slowness.” Additional preparation therefore functioned as a means of managing perceived inadequacy (19). Compensatory behaviors included working harder, increased efforts, studying in response to fear or embarrassment, seeking additional credentials and continually attempting to demonstrate professional legitimacy (21, 25, 27). In some studies, compensatory effort extended into daily routines such as using breaks to catch up with workload and continuing to work under sustained time pressure. However, the excessive workload was substantially driven by staffing and organization demands and was not particular to IP (20). In research context, repeated experimentation and prolonged troubleshooting following irreproducible research findings were identified which could disrupt productivity and, ultimately contribute to changing or abandoning research direction (28).

### 4.3 Performance capacity

#### 4.3.1 Theme 5: the subjective experience of incapacity

Across the included studies, performance capacity was represented predominantly through participants’ subjective experience of their own capacity, rather than through demonstrated objective impairment. Subjective experience of performance capacity was characterized by an interconnected combination of negative self-appraisal, self-questioning, perceived insufficiency and emotional distress. Participants frequently experienced themselves as inadequately knowledgeable, skilled or prepared for occupational demands, accompanied by feelings of anxiety, fear, inadequacy, panic, demotivation, frustration and uncertainty (18–21, 23–27). These perceptions persisted despite progression through training and increasing seniority; experienced simulation educators continued to question whether they were sufficiently qualified or capable of teaching, mentoring and occupying senior professional positions despite substantial professional experience and credentials (25). Participants reported feeling *“behind”* peers and lacking clinical or research skills. These perceptions were accompanied by vulnerability, alienation, dread and fear of evaluation (22). Self-doubt and lack of belonging were reported among women physicians with repeated discriminatory messages and questioning of their authority contributing to these perceptions (27). Taken together, the findings indicate that performance capacity in IP was represented predominantly through how capacity was experienced rather than evidence that capacity itself was impaired.

#### 4.3.2 Theme 6: how perceived incapacity interferes with doing

Although objective deficits in professional competence were not reported, a smaller group of studies described ways in which the experience of inadequacy could potentially interfere with occupational functioning, particularly learning, concentration, productivity and performance (20–23, 28). Attention and emotional energy was reported to be driven away from learning towards maintaining an appearance of competence, as one participant stated, “I wasn’t able to truly focus on learning. I was always more focused on the façade.” (23) One participant reported feeling drained and being unable to sleep. (23). Negative responses associated with IP could act as an *“insulating factor,”* preventing trainees from engaging in the reflection and perspective change necessary for transformational learning (21). Higher levels of stress and emotional overwhelm were reported to interfere with information processing and learning during transition among newly qualified nurses (20). However, exhaustion and difficulty keeping up with clinical responsibilities were reported alongside workload and occupational pressures, limiting attribution of these consequences specifically to IP. Emotional and cognitive responses could extend into broader functioning, such as following irreproducibility in research, participants reported rumination, difficulty concentrating and reduced productivity, while in some instances it interfered with sleep, eating, work and relationships. Somatic experiences including headache were also reported (28). Depression and anxiety symptoms were measured using the Patient Health Questionnaire-8 and Generalized Anxiety Disorder-7, however, the study did not establish that irreproducibility caused those symptoms (28). Participants described anxiety around evaluation, with one reporting that they would “psyche myself out” and perform worse. Another described stumbling and losing focus on relevant clinical information despite ordinarily following a systematic evaluation approach (22).

### 4.4 Environment

#### 4.4.1 Theme 7: relationships that validate v/s undermine professional legitimacy

The social environment emerged as a prominent contextual factor across studies, with relationships described as either reinforcing participants’ developing sense of competence (18–21, 24, 26, 28) or being associated with feelings of inadequacy or non-belonging (20, 22, 23, 27). Mentors, coaches and role models helped residents gain perspective on their abilities and challenge self-defeating thoughts. While peer sharing also helped normalize IP (19). senior clinicians were described as supporting residents’ perceptions of competence by normalizing uncertainty, sharing their own knowledge gaps, identifying strengths, supporting autonomy, publicly endorsing competence, and providing concrete feedback based on examples of successful performance, as reflected in, “telling patients they’re in good hands with me” and “empowering me in front of others by looking to me in front of patients or interns on rounds.” (24) Peers provided debriefing, reassurance, and recognition; while mentors, educators, managers, family and friends supported confidence and coping (20, 26), wherein supervision, preceptorship and supportive relationships were identified important during transitions and difficult clinical experiences (18, 21). In research context, participants described supervisors as shaping how unsuccessful findings were interpreted. Supervisors who considered alternative explanations, trusted student’s findings and validation of competence was described to support researcher identity (28).

Conversely, continually attributing unexpected findings to student error (28), constant comparison with peers, particularly with more experienced colleagues reinforced IP as participant questioned whether they would “ever be like this” when observing a senior resident confidently managing clinical responsibilities (23). Experiences of bullying, belittling, cliques, criticism, lack of trust, exclusion, negative comparison and inadequate senior support were described as problematic interpersonal influences (20, 22). Questioned competence, being talked over or ignored, differential treatment, microaggressions and exclusions among women physicians were also identified to reinforce IP (27), microaggressions challenged belonging, with one participant describing such experiences made them “wonder whether you are in the right place.” Other participants described having achievements invalidated by peers who attributed their success to diversity status (22).

#### 4.4.2 Theme 8: organizations that support development v/s amplify demands

The institutional and organizational environment operated in both supportive and problematic directions. Supportive environments provided structured supervision, feedback, opportunities for supported autonomy and resources for professional development (19, 24); whereas, problematic environments increased occupational demands through inadequate support, workload, evaluation pressures, poorly integrated training and expectations that exceed available resources (18, 20–23, 28). Formal biweekly meetings and structured opportunities to discuss IP, clear expectations, supportive feedback and supervised autonomy entrusting with patient-care decisions facilitated residents’ management of IP (19, 24). The organizational environment could therefore enable increasing competence without requiring unsupported independence (24).

Conversely, inadequate organizational support could impact increasing responsibility such as inadequate orientation, insufficient educator availability, staffing shortages, high patient loads and inadequate staffing ratios alongside participants feeling unprepared and overwhelmed (18, 20). Variation in support across workplaces further indicated that difficulties transitioning into practice could not be understood solely as an individual deficit. Educational and training structures could themselves become sources of pressure. Increasing responsibility and expectations for autonomy within an environment of continual evaluation and scrutiny, alongside uncertainty about expected knowledge contributed to perceived underperformance (23). Competency-Based Medical Education structures, including Entrustable Professional Activities and repeated workplace assessments, could produce assessment fatigue and administrative burden (21). Within the research context, qualifications, credentials, certifications, and measurable achievements operated as markers of competence (25). Publication pressures and supervisory control over research resources and direction were identified as contextual influences (28). Additionally, repeated transitions, poor programme integration, different expectations, limited resources and lack of mentors were also identified to reinforce IP (22).

#### 4.4.3 Theme 9: cultures of concealment, hierarchy and belonging

Across several studies, the broader professional culture communicated implicit and explicit expectations regarding what competent professionals should look like, how much uncertainty could be displayed and who was recognized as legitimately belonging (22–25, 27). Medical training is described to be characterized by hierarchy and expectations of expertise, certainty and confidence (23). Residents felt pressure to appear knowledgeable despite uncertainty, creating a discrepancy between internal experience and the professional image they believed they were expected to present. One resident explained, “there needs to be a certain level of confidence that comes out, but it might not match how you’re actually feeling.” (23), and another resident described, “culture of not addressing IP,” with some residents reporting burying their feelings as “faking until you make it,” whereas leaders who disclosed their own uncertainty and normalised IP demonstrated support (24). Healthcare simulation was described as hierarchical, with professional titles carrying assumptions of authority and competence; credentials and visible achievements could therefore become culturally embedded signs of legitimacy (25). Medicine was described as requiring confidence and knowledge, particularly around patients, while bias, microaggressions, social exclusion and othering further challenged legitimacy within both professional communities (22). Political and structural influences were inconsistently reported including gender inequality, sexism, racialized discrimination, power hierarchies and structural inequities shaped professional recognition and access to opportunities (27). Patriarchal and gendered expectations concerning who could legitimately occupy particular professional positions were also identified to contribute to impostor feelings. One participant rejected an individualized interpretation of these experiences, stating, “it’s not imposter syndrome, it’s you don’t want me in the field.” (22, 27).

Across the four MOHO domains, the findings demonstrated that IP is not confined to perceived competence or self-doubt but was expressed through interconnected occupational processes. Environmental feedback, expectations, recognition and social context were repeatedly described in relation to participants’ perceptions of competence and legitimacy within the volitional domain. These perceptions were, in turn, described alongside professional role development and role expectations within habituation, and were expressed through patterns of doing such as concealment, overpreparation and compensatory effort. Within performance capacity, participants described experiencing uncertainty, anxiety and perceived inadequacy during occupational performance despite demonstrating professional competence. Thus, the findings indicate that the four MOHO domains were interrelated in participants’ accounts rather than representing independent categories. The occupational interpretation of these relationships is considered further in the Discussion.

## 5 Discussion

Interpreted through MOHO, the findings suggest that IP among healthcare professionals is not simply characterized by persistent self-doubt but is experienced across interconnected occupational systems. Although all MOHO domains were represented, volition and environment were strongly represented, suggesting that perceptions of competence may develop through an ongoing interaction between personal and environmental contexts. Figure 2 illustrates this conceptualisation.

**Figure 2:**
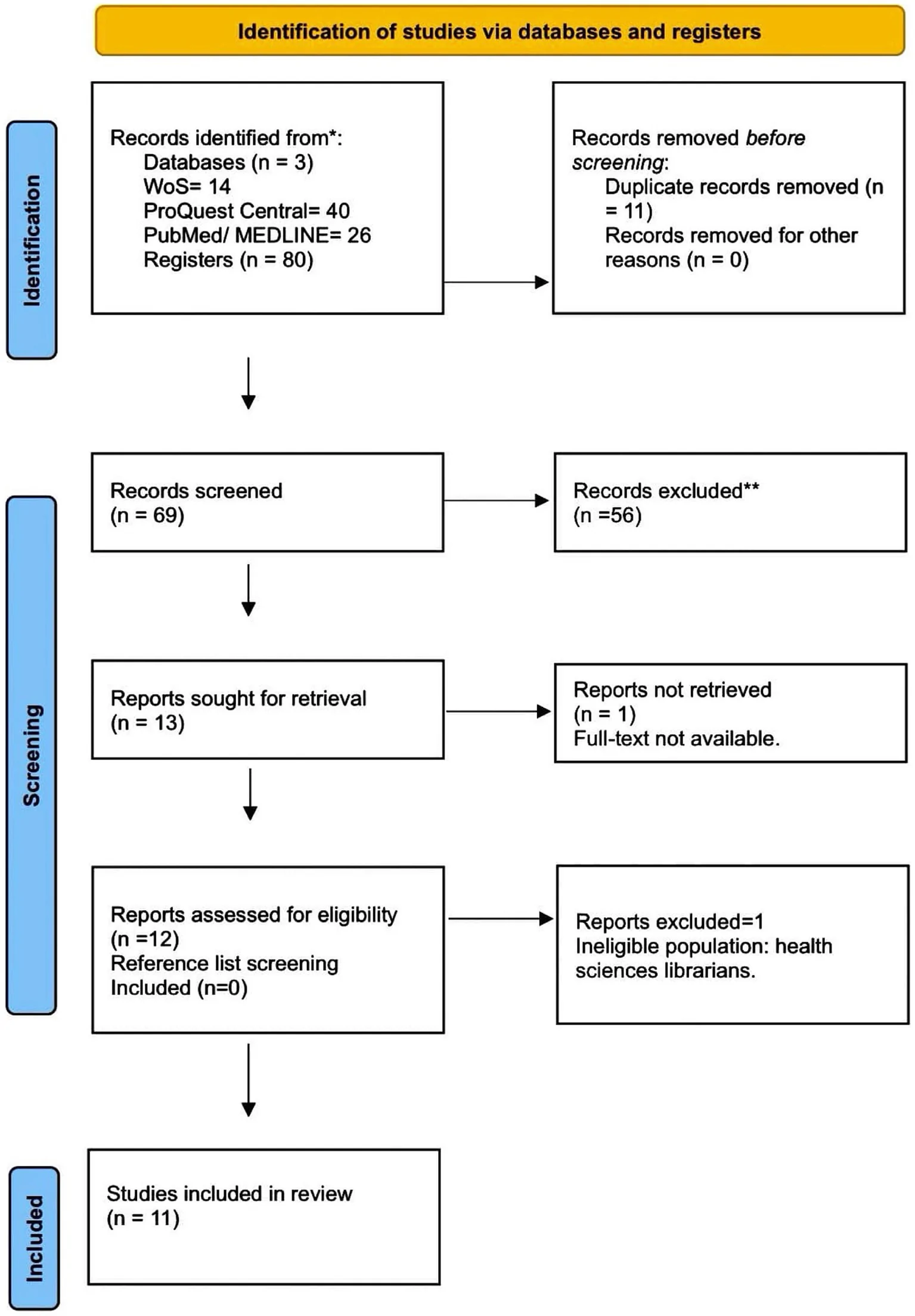
Conceptual representation of IP as an occupational experience through the MOHO. The figure illustrates the interconnections between volition, habituation and performance capacity. The outer environment encompasses and influences these domains through social, organizational and cultural contexts. Occupational adaptation is presented as an outcome reflecting the potential implications of these interconnected processes for occupational participation, performance and engagement.

Volition is a central determinant of occupational participation, influencing occupational choices, meaning and continued engagement in roles (29). The findings of the first theme described a process within personal causation, whereby healthcare professionals demonstrated competence while perceiving themselves as inadequate despite successful performance. This aligns with Clance and Imes, conceptualisation of IP, and Bravata et al. (30) findings that persistent self-doubt despite demonstrated achievements to be an important characteristic of IP (1). The present synthesis through MOHO extends these findings by suggesting that this discrepancy is maintained through occupational experiences, where achievements are discounted while mistakes are disproportionately interpreted as evidence of incompetence. Importantly, the discrepancy between demonstrated and perceived competence was not uniformly described as an internally generated process. Across the included studies, social evaluation, differential treatment and external questioning of competence also contributed to how participants interpreted their abilities. Consequently, these findings indicate that demonstrated participation and performance did not necessarily translate into a stable subjective sense of occupational competence. Whether this discrepancy persists over time requires longitudinal investigation.

Healthcare professionals consistently demonstrated strong professional values, including commitment to patient care, responsibility, lifelong learning and professional development, consistent with literature describing healthcare professionals as conscientious and achievement-oriented (3, 31). However, the present synthesis suggests that the valued occupations could become mechanisms for establishing or defending legitimacy. Patient care was associated with fear of harming others, professional development with proving competence, and achievement with defending anticipated failure. Within MOHO, values are convictions regarding what is meaningful, while interests generate enjoyment that sustain occupational engagement (13), together contributing to activity satisfaction influenced by experiences associated with successful completion of tasks (32). Thus, we propose IP may not necessarily reduce occupational participation but may alter its motivation and subjective experience behind participation, with valued occupations also serving to establish legitimacy, and avoidance of failure. Across these studies, competence and professional development appeared to remain valued even when participants questioned their ability to embody them. Together, the interpretation is that the volition disruption associated with IP was therefore not a loss of what individuals valued or wanted to do, but instability in their belief that they were capable of doing it successfully and legitimately.

Professional role acquisition was evident during transitions into senior or autonomous roles. The overall findings suggest that role transition may be particularly relevant to IP when becoming a senior or independent professional introduces expectations that have not yet been fully incorporated into occupational identity. Participants could assume increased responsibility while continuing to experience themselves as students, novices or outsiders, potentially interfering with the process of “occupational becoming” and professional identity formation. (30, 31). Repeated transitions and multiple professional roles could reactivate uncertainty regarding legitimacy.

Participants also adopted compensatory routines characterized by overpreparation, excessive checking, concealment of uncertainty and continual efforts to avoid perceived failure. The findings suggest that concealment was not merely an internal experience of fraudulence but could become a pattern of doing through which uncertainty was managed and kept from view. Similarly, avoidance of questions, visibility or help-seeking may have implications for opportunities for learning, support and professional participation. We suggest that these behaviors may reinforce the belief that competence depends on additional effort rather than capability. Within MOHO, habituation provides stability through adaptive roles and routines (13); these findings raise questions about occupational adaptation when professionals enact expected occupational roles while continuing to experience a disconnect between demonstrated performance and perceived competence. Therefore, these patterns raise questions about the sustainability of occupational engagement when additional effort, concealment and self-monitoring become recurrent features of professional participation.

Performance capacity was represented predominantly through the subjective experience of occupational performance rather than demonstrable objective deficits. Participants described persistent anxiety, self-doubt, fear of mistakes and perceived inadequacy despite successful performance. This is consistent with quantitative evidence linking IP with psychological distress, lower self-efficacy, and perfectionistic behaviors (3, 8). The included studies further indicated interference with concentration, learning, reflective practice and clinical decision-making, particularly during evaluation or increasing responsibility. Thus, from an occupational perspective, healthcare professionals may continue to perform occupations successfully while experiencing them through increased vigilance, exhaustion and self-monitoring. These experiences may have implications for reducing occupational satisfaction and the capacity for adaptation.

Although IP was originally conceptualized as an intrapersonal phenomenon, the present synthesis indicated that discounting achievement was not invariably a self-generated distortion but was also influenced by professional and sociocultural environment and shapes how competence, legitimacy and professional identity are experienced. Findings also suggest that difficulty inhabiting a professional role did not always arise from an individual’s inability to internalize it; the social environment could also fail to recognize their legitimacy within that role. Mentorship, psychologically safe feedback, trusted supervision and opportunities for reflection supported more realistic interpretation of occupational experiences. Conversely, hierarchy, continual evaluation, bullying, exclusion and discrimination reinforced self-doubt and feelings of illegitimacy. Similar associations between mentoring, supportive leadership and psychologically safe workplaces have been reported with reduced IP (31, 33). Within MOHO, occupational participation emerges through interaction with physical, social, cultural and organizational environments (11, 34). The present synthesis extends this perspective by suggesting that environments influence successful occupational participation as well as interpretation of performance.

The occupational reconceptualization presented in this scoping review refers to a conceptual expansion that may help shift the understanding of IP from an experience located primarily within the individual to one that can also be explored in relation to patterns of doing, professional role development, occupational participation and the environments in which these occupations happen. This review offers a broader understanding of how healthcare professionals perform, experience, interpret their occupations and develop as occupational beings. The MOHO synthesis brings together dimensions such as perceived competence, professional role development, patterns of doing, occupational participation, and the influence of environment and context, which are often considered separately in the IP literature. This conceptualisation complements the existing psychological understandings of IP by situating experiences of self-doubt and perceived inadequacy within the context of occupations and everyday participation. Mapping the findings to MOHO represents an interpretive synthesis rather than direct empirical testing of the model in context of IP. Discipline-specific databases such as PsycINFO and CINAHL, as well as broader databases such as Scopus and Embase, were not searched and may contain additional relevant literature. This represents a limitation of the search strategy and may have resulted in some relevant studies not being identified. The restriction to English-language publications may have resulted in the omission of relevant qualitative evidence published in other languages and may therefore have introduced language bias. The included studies came predominantly from high-income countries, which may limit transferability to other contexts. In addition, the included evidence was unevenly distributed across healthcare professions, with a substantial proportion of studies focusing on physicians, medical residents and medical trainees, while fewer studies represented nurses, allied health professionals and healthcare researchers. Therefore, although the synthesis identified occupational processes that may be relevant across healthcare roles, the extent to which these patterns may apply across the broader healthcare workforce cannot be assumed. The included studies were not critically appraised, as the primary objective of the scoping review was to map and thematically analyze the available qualitative evidence. Consequently, the differences in methodological quality among the studies were not assessed. Therefore, the synthesized themes reflect the patterns identified across the available evidence and not the assessment of the strength of the individual studies. Future research should examine occupational dimensions of IP across professions and career stages using occupation-centered frameworks, including longitudinal and intervention-based approaches to understand how IP may relate to occupational participation, role development, and patterns of doing over time. Research should also explore occupation-focused strategies within healthcare education and workplace environments, including approaches that support professional role development, perceived competence, and sustainable occupational engagement. Such work may help clarify how individual experiences of IP interact with occupational and environmental contexts and inform interventions that support healthier occupational development.

## 6 Conclusion

This scoping review provides an occupational perspective on the impostor phenomenon by synthesizing qualitative evidence through the Model of Human Occupation. Across the included studies, healthcare professionals described a persistent gap between demonstrated and perceived competence, while continuing to value patient care and professional growth. Transitions into senior or autonomous roles often coincided with feeling like a novice, and many participants relied on compensatory habits and routines such as overpreparation, self-monitoring, and concealing uncertainty. Performance was largely maintained but experienced through anxiety, vigilance and exhaustion, and social and organizational environments could either ease or reinforce IP. Interpreted through MOHO, we propose that IP may be understood as an occupational experience shaped by the dynamic person-environment interaction, although this proposition requires further cross-professional and longitudinal evidence. An occupational lens may inform education and workplace strategies that support role development, realistic appraisal of competence and sustainable patterns of engagement.

## Data Availability

The original contributions presented in the study are included in the article/Supplementary material, further inquiries can be directed to the corresponding author.
