# Supplementary material for: Reconceptualizing the impostor phenomenon as an occupational experience: a scoping review and framework synthesis of qualitative evidence guided by the model of human occupation
Source: Front Public Health. 2026 Oct 21;14:1975155. doi: 10.3389/fpubh.2026.1975155 (PMC13731273; doi:10.3389/fpubh.2026.1975155)
Supplement: Supplementary file 1 [file Table_1.DOCX]

Supplementary Material

**The supplementary tables provide the detailed search strategy, data extraction of included articles, and synthesis underpinning the findings of this scoping review.**

1.1 **Database-specific Search strategy**

**Supplementary Table S1: Database-specific search strategies**

| **Database** | **Search strategy** | **Filters/limits** |
| --- | --- | --- |
| PubMed/MEDLINE | *("impostor syndrome"[tiab] OR "imposter syndrome"[tiab] OR "impostor phenomenon"[tiab] OR "imposter phenomenon"[tiab] OR "impostorism"[tiab] OR "impostor feelings"[tiab]) AND ("health personnel"[MeSH] OR "healthcare professional*"[tiab] OR "health care professional*"[tiab] OR "health professional*"[tiab] OR physician*[tiab] OR doctor*[tiab] OR nurse*[tiab] OR pharmacist*[tiab] OR dentist*[tiab] OR resident*[tiab] OR "allied health"[tiab]) AND (qualitative[tiab] OR "qualitative research"[pt] OR "interviews as topic"[MeSH] OR "focus groups"[MeSH] OR interview*[tiab] OR "focus group*"[tiab] OR "thematic analysis*"[tiab] OR phenomenology*[tiab] OR "grounded theory"[tiab] OR "content analysis*"[tiab] OR "mixed-method*"[tiab] OR "mixed method*"[tiab])* | 10 years; English; Free full text |
| ProQuest | *(TI,AB("impostor syndrome" OR "imposter syndrome" OR "impostor phenomenon" OR "imposter phenomenon" OR impostorism OR "impostor feelings"))*  *AND*  *(TI,AB("healthcare professionals" OR "health care professionals" OR "health personnel" OR "health professionals" OR physicians OR doctors OR nurses OR pharmacists OR dentists OR residents OR "allied health" OR therapists OR "occupational therapists" OR "physical therapists"))*  *AND*  *(TI,AB(qualitative OR "qualitative research" OR interviews OR interview OR "focus group" OR "focus groups" OR phenomenology OR phenomenological OR "thematic analysis" OR "grounded theory" OR "content analysis" OR "mixed methods"))* | Scholarly journals; last 10 years; Articles; English |
| Web of Science | *("impostor syndrome" OR "imposter syndrome" OR "impostor phenomenon" OR "imposter phenomenon" OR impostorism OR "impostor feelings") AND ("healthcare professionals" OR "health care professionals" OR "health personnel" OR "health professionals" OR physicians OR doctors OR nurses OR pharmacists OR dentists OR residents OR "allied health" OR therapists OR "occupational therapists" OR "physical therapists" OR "medical students" OR "nursing students" OR "allied health students") AND (qualitative OR "qualitative research" OR interviews OR interview OR "focus group" OR "focus groups" OR phenomenology OR phenomenological OR "thematic analysis" OR "grounded theory" OR "content analysis" OR "mixed methods")* | Last 10 years; English; Article |

## 1.2 Supplementary Table 2: Data extraction from included studies

| **S. no**. | **Author, year** | **Country** | **Profession** | **Aim of the study** | **Methodology** | **Key findings** |
| --- | --- | --- | --- | --- | --- | --- |
| 1. | Mirabal et al., 2024 (19) | USA | Internal medicine residents (n=28) | To identify the factors that mitigate impostor phenomenon among internal medicine resident. | Qualitative study, semi-structured telephone interview and constructivist thematic analysis. | IP was described as a cyclical process involving feelings of inadequacy, avoidance behaviours, distorted interpretation of positive feedback and maladaptive attribution beliefs. Increasing clinical responsibility, uncertainty and fear of being exposed intensified IP. Protective factors included reframing attribution beliefs, accepting feedback, recognising personal strengths, building self-confidence and adopting a growth mindset; along with supportive mentors, role models, sharing of impostor experiences, structured discussions and psychologically safe learning environments. |
| 2. | Chodoff et al., 2023 (23) | USA | Internal medicine residents (n=28) | To explore how IP is experienced by internal medicine residents during training | Semi-structured interviews with thematic analysis. | IP is described by residents as a pervasive feeling of “not being good enough”, often reinforced by comparison with peers. Role transitions, increasing responsibility and expectations of autonomy, constant scrutiny, and medical hierarchy contributed to IP. Residents struggled to internalize positive feedback, attributed success to luck and developed unrealistic expectations of their competence and responsibility for patient outcomes. IP hindered learning by increasing anxiety, limiting vulnerability and maintenance of appearance of competence. Supportive coaching, constructive feedback and reframing performance situations as opportunities for facilitating learning facilitated growth. |
| 3. | LaDonna et al., 2025 (27) | Canada | Women in medicine (N=40). Faculty physicians (n=13), residents (n=14), and medical students (n=13). | To examine the relationship between gender-based discrimination and imposter feelings of women in medicine. | Constructivist grounded theory using semi structured interviews and constant comparative analysis. | Complex relationship was described between IP and gender-based discrimination; some participants perceived no association, while others described discrimination as triggering or intensifying self-doubt. Gender bias, exclusion, differential standards, and intersectional discrimination led some women to question their competence and belonging, which others recognised these as externally imposed. Experiences such as gaslighting contributed to hypervigilance, pressure to continually prove competence, and reduced feelings of belonging. Authors developed a concept of the Intruder Paradox (IPx) to describe externally imposed perceptions of incompetence and alienation directed toward individuals who do not fit established professional norms. IPx though distinct may co-exist with IP. |
| 4. | Freeman et al., 2025 (25) | International.  Sample from Oceania, North America and Europe.  Research team based in Australia | Healthcare simulation educators from multiple discipline (N=20): Nursing (n=7), medicine (n=3), physiotherapy (n=2), pharmacy (n=1), paramedicine (n=1), and unspecified/other profession (n=6). | To explore the lived experiences of IP among healthcare simulation educators and understand its influence on their careers. | Hermeneutic phenomenological study using semi-structured interviews and iterative interpretive analysis. | Lived experience of IP were described with four themes. First, reflected feeling of being insufficiently qualified and a continued need for credential and external validation. Second, described self-minimisation, including reduced professional visibility, reluctance to speak up and missed opportunities. Third, reflected the dual nature of IP, which could motivate professional growth while also contributing to insecurity, perfectionistic striving and exhaustion. Lastly, fourth theme captured the cyclical recurrence of self-doubt during new roles, responsibilities and professional challenges. IP consequently influenced professional identity, career development and well-being. |
| 5. | Ryan et al., 2025 (20) | Australia | Early career registered nurses within first 2 years of professional practice (N=26) | To explore the lived experience of transitioning from student to early-career nurse while navigating the workplace. | Qualitative exploratory study informed by hermeneutic phenomenology; semi-structured interviews and thematic analysis. | Three themes were identified. The first theme reflected feelings of being an imposter, inadequate, overwhelmed and ill-prepared for independent practice. The second described high workloads, staffing shortages, inadequate support, workplace bullying and pressure which undermined confidence, constrained learning, and contributed to intentions to leave profession or workplace. The third theme highlighted the importance of support from fellow early-career nurses, mentors, educators, managers family and friends in coping with the transition. |
| 6. | Rivera et al., 2021 (26) | USA | Health professionals and workers (N=92) including residents, fellows, staff, faculty and institution leadership. Distribution not reported. | To discuss the impact of IP and develop strategies to confront IP at individual, peer and institutional level. | Educational workshop including didactics, individual reflection, and group discussion. Evaluated using a post-workshop survey; quantitative responses analysed descriptively and open-ended responses analysed using content analysis. | Workshop was found to meet its learning objective, was reported as a valuable use of time and participants intended to apply what they had learnt. Strategies to address IP were identified at three levels. At individual level, self-compassion, mentorship, vulnerability and recognition of accomplishments were noted. The peer level included celebrating colleagues’ successes and helping others recognise IP, and at the institutional level awareness and education about IP was recognised. Barriers included self-doubt and ingrained thought patterns, limited peer support and openness, difficulty changing institutional culture and workload pressures. |
| 7. | Gupta et al., 2025 (24) | USA | Residents (N=125)  Internal medicine (n=87), general surgery (n=20) and obstetrics-gynaecology residents (n=18) | To identify factors associated with IP and explore resident’s perspective on management of IP through team leadership | Mixed-method cross-sectional survey; qualitative analysis with hybrid inductive-deductive coding and theme development. | Four leadership strategies were identified as helpful in managing IP. First, normalising IP and sharing of IP experiences by leaders helped validate residents’ feelings. Second, constructive feedback which helped recognise strengths and identify areas for improvement. Third, demonstrating trust through autonomy and support strengthened confidence. Fourth, creating a safe space to discuss negative thoughts with mentors to challenge negative self-perceptions and reinterpret mistakes. Concealment and independent management of IP were also described. |
| 8. | Lubega et al., 2023 (28) | USA | Biomedical science doctoral students; survey (N=80), with semi-structured interview (n=37) | To examine how frequently biomedical doctoral students experience irreproducibility, how they respond to these experiences and their perceived impact on mental health. | Mixed-method study using an online survey followed by semi-structured interviews. Survey data were analysed descriptively and qualitative interview data using content analysis. | 84% participants were reported to fail to replicate their own results, 70% a colleague’s work and 58% published findings. Students commonly initially attributed irreproducibility to their own error or lack of skill, contributing to self-doubt and loss of confidence; some explicitly described these experiences as triggering impostor feelings. Emotional responses included frustration, depression, demotivation and anxiety, sometimes interfering with daily functioning. Supportive mentorship helped reframe self-blame and validate research competence, whereas attribution of irreproducibility to student error by supervisors reinforced self-doubt and impostor feelings. |
| 9. | Ford et al., 2024 (18) | UK | Newly qualified mental health nurses within 18 months of registration from two NHS mental health trusts (N=18). | To understand newly qualified mental health nurses’ experience of transition and its potential impact on remaining in the role. | Grounded theory based qualitative exploratory study with a constructivist perspective; semi-structured interviews analysed using thematic content. | Five themes described transition experiences. Transition to the new role involved increased responsibility, reality shock, anxiety and feeling overwhelmed. Formal supervision, preceptorship and informal team support facilitated transition, although support was considered inconsistent. Developing professional identity involved adjusting to being recognised and seeing oneself as a competent nurse alongside changing expectations. Role development involved challenges such as self-pressure and imposter phenomenon, as well as leadership and organisational demands. The working environment presented high workload, staffing shortages and safety concerns. |
| 10. | Chow et al., 2024 (21) | Canada | Cardiology residents and recent graduates (N=22); including residents from fourth-year (n=6), fifth-year (n=5), sixth-year (n=4) and recent graduates (n=8). | To explore the influence of transformational learning experiences on professional identity formation within a competency-based medical education framework (CBME). | Theory-informed inductive qualitative study using semi-structured interviews; data analysed using reflexive thematic analysis informed by transformational learning theory. | CBME showed limited connection with transformational learning. Clinical encounters and mentor relationships facilitated transformational and professional identity formation. Impostor phenomenon was reported in nearly all interviews, particularly earlier in training, and involved self-doubt, fear of mistake or embarrassment, and concerns about appearing incompetent. It was reported that fear of poor performance could motivate greater effort, while persistent self-directed negativity associated with IP could act as an “insulating factor” preventing challenging experiences from producing transformation. Unsolicited or non-credible feedback acted as an identity threat that could inhibit transformation. |
| 11. | Chakraverty et al., 2022 (22) | USA | MD-PhD trainees.  Participants completed survey (n=13) and interview (n=9). | To explore reasons and factors contributing to impostor phenomenon among MD-PhD trainees particularly those from underrepresented groups. | Mixed-method study using an online survey with CIPS followed by semi-structured interviews; qualitative data analysis using constant comparative method and analytic induction. | Four interconnected themes described IP. First was professional identity formation where trainees struggled to develop a physician-scientist identity and lacked a sense of belonging in medicine or research. Second was fear of evaluation by faculty, peers and patients which contributed to self-doubt and concerns about competence. Third was minority status wherein first-generation background, bias, microaggressions and social exclusion contributed to alienation and IP. Fourth was transitions between medical and research training programme which created stress, feelings of being unprepared and concerns about competence. Mentoring and inclusive learning environments were identified as important for supporting professional identity formation and addressing IP. |

**1.3 Supplementary Table 3: Framework matrix charting of extracted findings across MOHO domains**

| S. no. | Author, year | Volition | Habituation | Performance capacity | Environment |
| --- | --- | --- | --- | --- | --- |
| 1. | Mirabal et al., 2024 (19) | **Personal causation:** Persistent inadequacy and self-doubt; difficulty internalising competence and positive feedback; external attribution of success.  Reframing attribution beliefs, accepting feedback and recognising strengths supported confidence and recognition of competence.  **Values**: Sense of responsibility towards patients was evident but indirectly represented. **Interests**: Not identified. | **Roles**: Increased expectations and clinical responsibilities challenged perceived competence in fulfilling the role of a resident/ physician. **Habits**: Avoidance of asking questions or seeking help pretending to know and compensatory overpreparation. **Routines**: Excessive preparation, arriving early and working additional hours to compensate for perceived inadequacy. | **Subjective capacity**: Recurrent thoughts and feelings of inadequacy, fraudulence, self-doubt, fear of exposure, self-defeating thought processes, uncertainty and reduced confidence; perceived unpreparedness for clinical tasks and responsibilities despite expectations of competence. **Objective capacity**: No objective impairment in clinical ability was reported. | **Social**: Mentors, coaches, role models and peer sharing provided reassurance, perspective and normalisation of IP thoughts. **Institutional**: Formal support opportunities and supportive, collaborative, growth-oriented learning environments mitigated IP. **Cultural**: Performance-focused expectations and learning cultures influenced IP, while acceptance of uncertainty, mistakes and seeking help supported residents. **Physical**: Not identified. |
| 2. | Chodoff et al., 2023 (23) | **Personal causation:** Persistent feelings of inadequacy and fraudulence, self-criticism, low self-confidence and doubts regarding knowledge and competence. Residents compared their performance with others, interpreted mistakes as evidence of inadequacy, discounted positive feedback and attributed achievements to luck. **Values**: Strong responsibility for patient outcomes was evident, with residents assuming disproportionate personal responsibility when outcomes were poor. Interests: Learning and professional growth were desired, but attention was diverted from learning towards proving competence. | **Roles**: Role transitions, increasing clinical responsibility, greater autonomy and expectations to provide answers challenges residents’ confidence in fulfilling senior professional roles. Habits: Impression management and projecting confidence despite internal uncertainty; concealment of vulnerability; persistent comparison with peers; focusing on appearing competent rather than exposing learning needs. **Routines**: IP recurred particularly at the beginning of new clinical rotations and during transitions to greater responsibility. | **Subjective capacity:** Intense self-criticism, inadequacy, fraudulence, anxiety, fear, apprehension, insecurity, feeling overwhelmed and inadequately prepared, and difficulty processing feedback. One participant described feeling drained and being unable to sleep following difficult patient experience. **Objective capacity**: No objective deficit in clinical competence reported, residents felt feelings of incompetence persisted despite experience and evidence of adequate performance. | **Social**: Constant comparison with peers, scrutiny by others, interactions with nurses and expectations from junior residents influenced IP; constructive feedback coaching could facilitate reflection and growth. **Institutional**: Increasing responsibilities, expectations of autonomy, unclear expectations regarding required knowledge, performance-focused training and constant evaluation intensified IP. **Cultural**: Rigid medical hierarchy, expectations of certainty or expertise and pressure to project confidence and suppressing of vulnerability perpetuated IP. Physical: Not specifically identified. Different rotations such as ICU and other services were contexts in which IP occurred but physical characteristics of these environments were not reported. |
| 3. | LaDonna et al., 2025 (27) | **Personal causation**: Self-doubt and questioning of competence and belonging were often shaped by externally imposed perceptions of incompetence, discrimination and gaslighting; some participants resisted these messages and maintained confidence in their competence. **Values**: Resistance to problematic gendered and sociocultural expectations was reported, although personal occupational values were not reported. **Interest**: Not reported, although career aspirations and professional opportunities were identified. | **Roles**: Gendered expectations and exclusion challenged recognition and enactment of the professional role, with women pressured to conform to expected professional identities. **Habits and routines:** Hypervigilance, constant self-monitoring, altering performance to fit in, repeatedly proving competence and sometimes not speaking up for opportunities. | **Subjective capacity**: Self-doubt, inadequacy, discomfort, feeling unworthy, lack of belonging and questioning of competence, repeated discrimination generated accumulated doubt and was sometimes described as a trauma response. Hypervigilance and constant self-monitoring increasing psychological demands. **Objective capacity**: No objective impairment reported. | **Social**: Competence was questioned or second-guessed through interactions with colleagues, supervisors, assessors, patients and other professionals. **Institutional**: Differential standards, inadequate support and barriers in hiring, leadership and career advancement. **Cultural**: Patriarchal and gendered professional norms, misogyny and pressure to conform shaped competence and belonging. **Political**: Sexism, racism, intersectional discrimination, power hierarchies and structural inequities contributed to exclusion and externally imposed incompetence. **Physical**: Not specifically identified. |
| 4. | Freeman et al., 2025 (25) | **Personal causation**: Persistent self-doubt, inadequacy and perceived lack of legitimacy despite experience and accomplishments; difficulty internalising credential or external validation and recurrent need to prove worth. IP could also motivate self-improvement and professional growth. **Values**: Professional legitimacy, integrity, competence and continuous improvement were evident, although, these were reported to be partly shaped by professional expectations. **Interests**: Limited evidence, learning and professional growth were described but specific interests were not explored. | **Roles**: Transitions between teacher, facilitator, learner, clinical and senior roles could increase IP and challenge professional identity. **Habits and routines:** Self-minimisation, silence, withdrawal, reduced contribution in meetings, avoidance of visibility or opportunities, repeated credential seeking, overworking and striving to prove competence became recurring patterns of doing. | **Subjective capacity**: Persistent self-doubt, inadequacy, insecurity, anxiety, fear of failure and exposure, second-guessing and doubts about belonging and professional ability. Continual striving and self-monitoring created psychological demands and emotional exhaustion; IP was described as draining and could compromise well-being. **Objective capacity**: No objective professional impairment reported despite participant’s perceived inadequacy. | **Social**: Comparison with highly credential peers and status attached to professional titles influenced legitimacy and belonging; external validation provided temporary reassurance. **Institutional**: Emphasis on credentials, qualifications, measurable achievement and expectations of expertise reinforced IP. **Cultural**: Hierarchical professionals culture and visible markers of success shaped perceptions of legitimacy and competence. **Physical**: Not identified. |
| 5. | Ryan et al., 2025 (20) | **Personal causation**: Inadequacy, self-doubt, low confidence and persistent questioning of ability to provide appropriate care; successful clinical experiences, mentor affirmation and positive patient feedback reinforced competence. **Values**: Safe, meaningful patient care and making a difference were valued, inability to provide desired care caused distress. **Interests**: Satisfaction from patient care and positive patient interactions supported continued engagement with nursing, although specific interests were not directly explored. | **Roles**: Transition from dependent student to independent registered nurse created tension as early-career nurses assumed decision making and patient-care responsibility while still feeling unprepared. **Habits and routines:** Repeated self-questioning, seeking help and peer debriefing; coping by *“going with the flow”.* High workload altered routines through working while rushed and using breaks to catch up. | **Subjective capacity**: Perceived unpreparedness and gaps in clinical knowledge; constant second-guessing, inadequacy, fear, anxiety, stress, overwhelm and emotional distress. High stress interfered with learning and information processing. **Objective capacity**: Constant pressure, exhaustion, excessive workload and time constraints contributed to physical discomfort. Cognitive and clinical difficulties were participant-reported. | **Social**: Peer support, mentors, educators, nursing managers, family and friends facilitated coping and confidence, while bullying, belittling and inadequate support undermined confidence. **Institutional**: Inadequate orientation and training, staff shortages, high workloads, limited educator availability, insufficient staffing/support intensified IP and overwhelm. **Cultural**: Bullying, hierarchy and unhealthy norms around breaks, sickness and prioritising patient care contributed to strain. **Physical**: Clinical setting influenced demands and access to support, workforce and resource constraints were reported, but physical characteristics were not directly reported. |
| 6. | Rivera et al., 2021 (26) | **Personal causation:** Self-doubt and entrenched negative though processes were barriers to confronting IP; participants intended to use self-compassion and reminders of accomplishments or positive evaluations to reinforce recognition of competence. **Values**: Support for peers, celebration of others’ successes and promotion of inclusion were indirectly reflected. **Interests**: Not identified. | **Roles**: Limited evidence; participants occupied multiple professional roles and intended to address IP through individual, peer and institutional level actions. Difficulties related to specific roles were not directly reported.  **Habits and routines**: Entrenched patterns of thought were identified as barriers, intended behavioural changes included seeking mentorship, practising self-compassion, allowing vulnerability, sharing IP experiences and supporting peers. | **Subjective capacity**: Self-doubt and ingrained negative thought processes were identified as barriers, with stress and disappointment facilitating recurrence of IP. Accomplishments and positive evaluations were proposed as reminders against perceived inadequacy. **Objective capacity**: Not reported. | **Social**: Mentorship, peer celebration and openly sharing impostor experiences were identified as supportive strategies; lack of peer support and openness were barriers. **Institutional**: IP education and awareness was proposed, while workload pressures, lack of viable avenues for institutional input, and difficulty changing institutional culture were barriers. **Cultural**: Openness could promote a supportive culture, whereas entrenched departmental culture impeded change. |
| 7. | Gupta et al., 2025 (24) | **Personal causation**: Feelings of inadequacy despite demonstrated competence. Specific feedback, recognition of strengths, demonstrated trust and supported autonomy reinforced confidence and self-efficacy. **Values**: Responsibility toward patient care was indirectly reported, particularly around mistakes, but occupational values were not directly reported. **Interests**: Not identified. | **Roles**: Normalising knowledge gaps reinforced the resident’s learner role, while autonomy, leading patient care, decision-making and visible recognition by leaders supported the developing physician and team-leader role. **Habits and routines:** Concealment of IP, independently managing negative feelings and *“faking until making it”* were recurring patterns, some residents reported burying and hiding IP almost daily. Open discussion, feedback-seeking and supported checking of clinical reasoning represented alternative patterns. | **Subjective capacity**: Self-doubt, inadequacy, uncertainty, negative self-perceptions, feeling undeserving and perceived lack of knowledge; mistakes could be disproportionately personalised. Leaders helped residents reinterpret negative thoughts through concrete evidence of competence and shared or system-level responsibility for mistakes. **Objective capacity**: Participants demonstrated good clinical and leadership performance despite perceived inadequacy. | **Social**: Normalisation, leaders sharing their own impostor feelings, specific constructive feedback, recognition of strengths, trust, mentorship and safe conversations helped manage IP. **Institutional**: Supported autonomy and clinical team structures that allowed residents to lead while retaining senior support reinforced competence. **Cultural**: Concerns are described as a *“culture of not addressing IP”*, particularly among surgical specialities and encouraged concealment. Psychological safety facilitated openness. **Physical**: Not identified. |
| 8. | Lubega et al., 2023 (28) | **Personal causation:** Irreproducibility triggered self-blame, self-doubt, loss of confidence and questioning of research competence, with students initially attributing unexpected findings to their own lack of knowledge, skill or error. Successful troubleshooting, experience and supportive mentorship helped restore confidence. **Values**: Scientific rigour and recognising rigorously obtained findings as valuable, even when unexpected, appeared as a protective factor. **Interests**: Motivation and enthusiasm for research could decline following repeated irreproducibility. | **Roles**: Irreproducibility challenged the developing researcher identity, particularly among less experienced doctoral students; accumulated experience and successful projects strengthened identity. **Habits**: Self-blame, repeated checking and troubleshooting were common responses. **Routines**: Repeated experimentation disrupted research activities and productivity; some participants shifted research questions or abandoned lines of research. | **Subjective capacity**: self-doubt, loss of confidence, frustration, demotivation or depressive feeling, anxiety and panic; participants questioned their research ability and experienced rumination and difficulty concentrating. Emotional responses could interfere with concentration, productivity, work, sleep, eating and relationships. **Objective capacity**: Depression and anxiety were assessed using PHQ-8 and GAD-7, but their associations with irreproducibility were analysed to infer causation. Sleep disturbances, altered eating and headaches were reported physical discomforts. | **Social**: Supportive supervisors validated competence, suggested alternative explanations and strengthened research identity, whereas supervisors attributing problems to student error reinforced IP. **Institutional**: Publication pressures, supervisory direction and doctoral training structures shaped responses to irreproducibility. **Cultural**: Expectations surrounding successful reproducible results and trust in published literature contributed to self-blame; while experience enabled more critical interpretation of unexpected findings. **Physical**: Laboratory context was present but physical environmental characteristics were not reported. **Political/structural**: Supervisory power over resources, research direction and asymmetry in student-advisor relationships were reported. |
| 9. | Ford et al., 2024 (18) | **Personal causation:** Feeling unprepared, reduced confidence and uncertainty regarding competence during transition into practice; experience, successful clinical encounters and support contributed to developing confidence. **Values**: Concern for safe and appropriate patient care and meeting professional expectations were indirectly reported. **Interests**: Not identified. | **Roles**: Transition from student to newly qualified nurse involved increased responsibility and accountability, with difficulty adjusting to and identifying with the professional nurse role. **Habits**: seeking help and reliance on supervisors accompanied recurrent self-questioning in unfamiliar situations. **Routine**: Adjustment involved managing increased responsibilities, workload and everyday clinical demands; consistent supervision supported adaptation. | **Subjective capacity**: Feeling unprepared, self-doubt, uncertainty about knowledge and clinical ability, reduced confidence, anxiety and feeling overwhelmed in response to increased responsibility and unfamiliar demands. **Objective capacity**: No objective impairment in competence reported. | **Social**: Supervision, preceptorship, team support and supportive colleagues facilitated confidence and transition, while inconsistent support hindered adjustment. **Institutional**: Staffing shortages, workload pressures, inconsistent preceptorship and workplace expectations shaped transition experiences. **Cultural**: Expectations regarding what newly qualified nurses should know and be able to do contributed to pressure and perceived unpreparedness. |
| 10. | Chow et al., 2024 (21) | **Personal causation**: Recurring self-doubt and uncertainty regarding competence and performance; trainees feared performing below expectations, making mistakes and appearing incompetent despite advancing through training. **Values**: Commitment to patient care and honouring patient’s and mentors’ trust was reported. **Interest**: Not identified. | **Roles**: IP persisted during development of the cardiologist identity, despite increasing seniority, skills and responsibilities. **Habits and routines:** Fear of poor performance and embarrassment motivated working hard to avoid mistakes and appearing incompetent. | **Subjective capacity**: Recurring self-doubt, uncertainty about performance, fear of mistakes, embarrassment and appearing incompetent; persistent self-directed negativity. Negative emotional responses with IP insulated trainees from reflection and transformational learning. **Objective capacity:** Not reported. | **Social**: Mentor-mentee relationships, trust, sponsorship and supportive interpersonal relationships enabled professional development; discussion with peers helped reinterpret difficult clinical experiences. **Institutional**: Competency-based Medical Education assessment requirements, assessment fatigue and administrative burden could interfere with authentic feedback and learning opportunities. **Cultural**: Performance expectations and concern with appearing knowledgeable and competent were reported. Unsolicited or non-credible feedback acted as an identity threat. |
| 11. | Chakraverty et al., 2022 (22) | **Personal causation**: Participants questioned their competence in medicine as well as research, felt less capable than their peers, discounted their achievements and perceived self as underserving of recognition. **Values**: Challenges around integrating patient care and research within a physician-scientist role. **Interests**: Mismatch between patient-focused research interest and basic-science research training. | **Roles**: Difficulty developing and internalising the dual physician-scientist identity; participants could feel they did not fully belong in either medicine or research. **Habits**: Hiding perceived weakness, reluctance to ask questions, negative peer comparison and second-guessing were reported. **Routines**: Repeated transitions between medical and research phases required adapting to different expectations and relearning knowledge, disrupting established patterns of functioning. | **Subjective capacity:** Feelings and thoughts of inadequacy, incompetence, anxiety, stress, alienation and fear of evaluations. Participants perceived gaps in clinical and research ability and preparation. Evaluation anxiety could disrupt performance, including loss of focus and poorer performance. **Objective capacity**: Not reported. | **Social**: Peer comparison, exclusion, faculty and patient evaluation, bias and microaggressions contributed to self-doubt, alienation and IP; lack of appropriate support and mentorship was also reported. **Institutional**: Repeated transitions and poor integration between research and clinics, differing expectations and limited resources contributed to difficulty adapting. **Cultural**: Distinct cultures of medicine and research, expectations to appear confident or knowledgeable and experiences of othering affected belonging. **Political/structural:** Underrepresentation, systemic bias and differential treatment provided structural context for participants’ experiences. |
